# A Rare Occurrence of Bilateral Ureteral Presence Within Inguinal Hernias in a Military Veteran: A Case Report

**DOI:** 10.7759/cureus.70831

**Published:** 2024-10-04

**Authors:** Brenden E McQueeney, Michael Breiner

**Affiliations:** 1 Medicine, Edward Via College of Osteopathic Medicine, Blacksburg, USA; 2 Surgery, Edward Via College of Osteopathic Medicine, Blacksburg, USA

**Keywords:** abdominal anatomy, bilateral, genitourinary anatomy, inguinal hernia, ureters

## Abstract

Anatomical disruption of the ureter is a rare phenomenon that alters the positioning of the ureter within the abdominal or pelvic cavity. It is even rarer for ureteral presence to be located within inguinal hernias, especially bilaterally. We explore a case of a 76-year-old male with bilateral inguinal hernias, each containing a portion of the ureter from its respective kidney. The patient initially presented to the internal medicine service with hematochezia secondary to diverticulitis and was largely asymptomatic from the perspective of genitourinary function. The patient’s presenting condition was resolved with appropriate antibiotic treatment, and the ureteral findings were used for patient counseling and education on the risks of future hernia surgery, should the patient need it. This report will present the relevant imaging findings that highlight the anatomy in question, as well as discuss possible etiologies associated with this anatomical finding. We will also explore the risks that pose a threat to ureters within an inguinal hernia and highlight the importance of thorough preoperative and intraoperative guidance and visualization to ensure no ureteral damage has occurred. This case serves as an example of the potential risks to be aware of prior to performing a routine surgical procedure and highlights a potential need for more reliability and comfort with medical imaging modalities.

## Introduction

Ureters are two anatomical structures found within the retroperitoneum of the abdominal cavity. They transport urine produced in the kidneys to the bladder for storage prior to excretion. The typical course of each ureter is to descend from the kidney to the bladder starting from its origin at the ureteropelvic junction, which lies posterior to the renal vein and artery. The ureter then descends along the anterior surface of the psoas muscle until reaching the bladder, concluding at the ureteral orifices of the bladder trigone [1,2].

Inguinal hernias are very common, both in the United States and worldwide. They affect up to 40% of men and 5% of women throughout their lifetime and are one of the most frequent problems treated by a general surgeon [3]. Inguinal hernias are commonly repaired surgically, but clinical monitoring has also been shown to be an effective option [4]. They typically present as a lump or mass containing bowel or adipose tissue found within the groin, which can range symptomatically from moderate discomfort to severe pain. Hernias tend to be reducible, which is the ability to apply pressure on the mass to return the contents to the abdominal cavity. Once the hernia can no longer be reduced but has adequate blood supply, it is then termed incarcerated. One of the major risks associated with inguinal hernias is strangulation, which occurs when the blood supply to the herniated contents is lost and ischemia ensues [5]. When the blood supply is cut off to the structures contained within the hernia, the presence of a ureter within the hernia could result in life-threatening complications.

Ureters within an inguinal hernia are a rare and potentially dangerous finding. It has been noted that fewer than 10 cases of ureteral inguinal hernias have been reported among adults with native kidneys, and the majority occur in patients undergoing renal transplants. This herniation could pose potential obstructive complications, such as renal failure, hydronephrosis, or urinary symptoms [6-8]. Ureteral presence within an inguinal hernia can be diagnosed with imaging modalities such as ultrasound, magnetic resonance imaging, or computed tomography during preoperative planning [9,10]. Although ultrasound is the first-line imaging modality, ultrasound-based diagnosis may miss the presence of a ureter within an inguinal hernia due to the varying skill levels of those performing ultrasounds. The patient featured in this case was largely asymptomatic from the perspective of genitourinary function. However, this case highlights the key importance of imaging and clinical evaluation of anatomic structures contained within inguinal hernias prior to surgical herniorrhaphy as a means of preventing ureteral injury. This unique case explores a 76-year-old male who initially presented to the hospital with hematochezia and was then found to have asymptomatic bilateral ureteral presence within inguinal hernias on imaging.

## Case presentation

**Figure 1:**
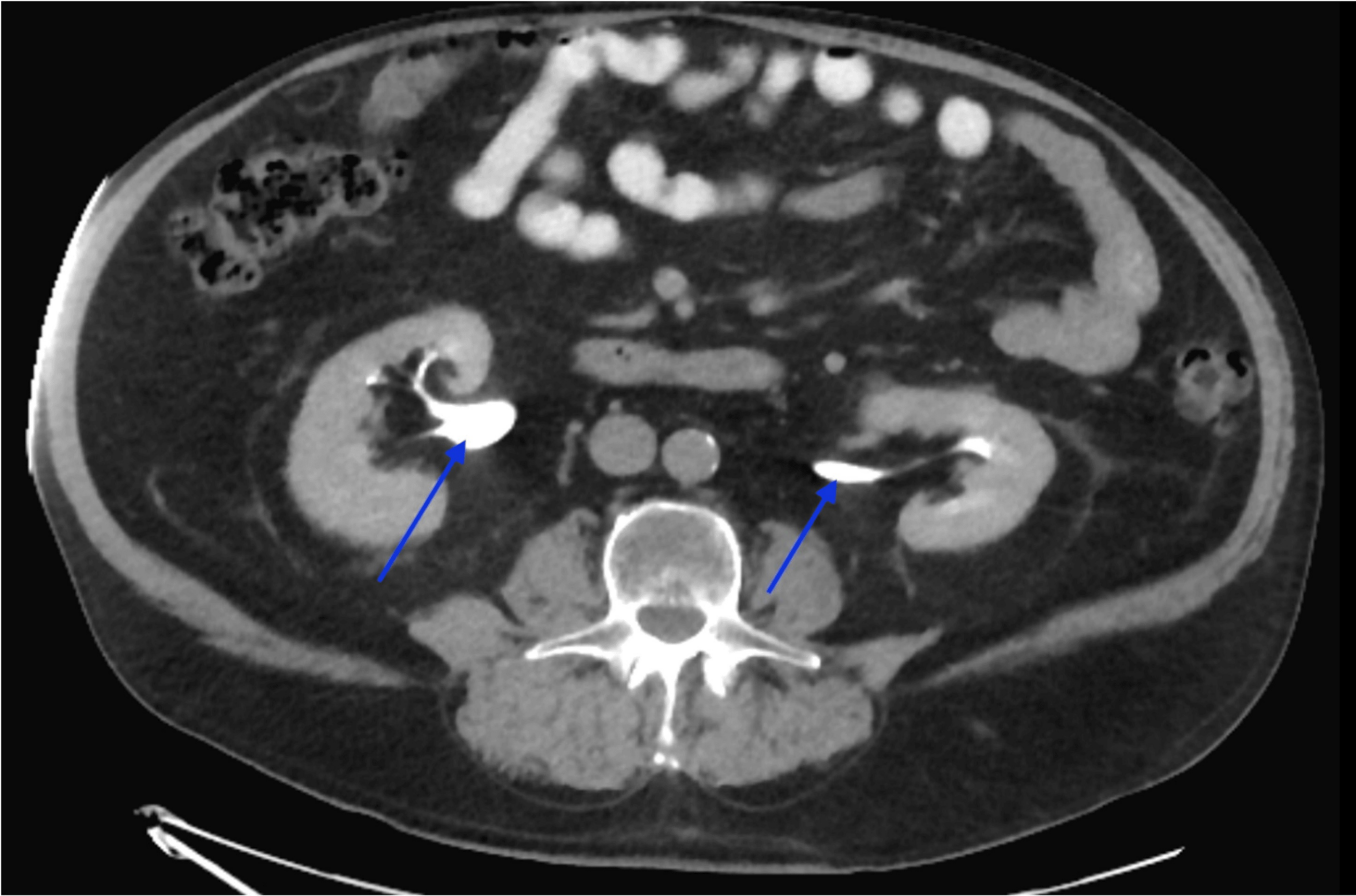
CT with contrast showing the ureters’ origin (blue arrows) at the patient's renal pelvis.

**Figure 2:**
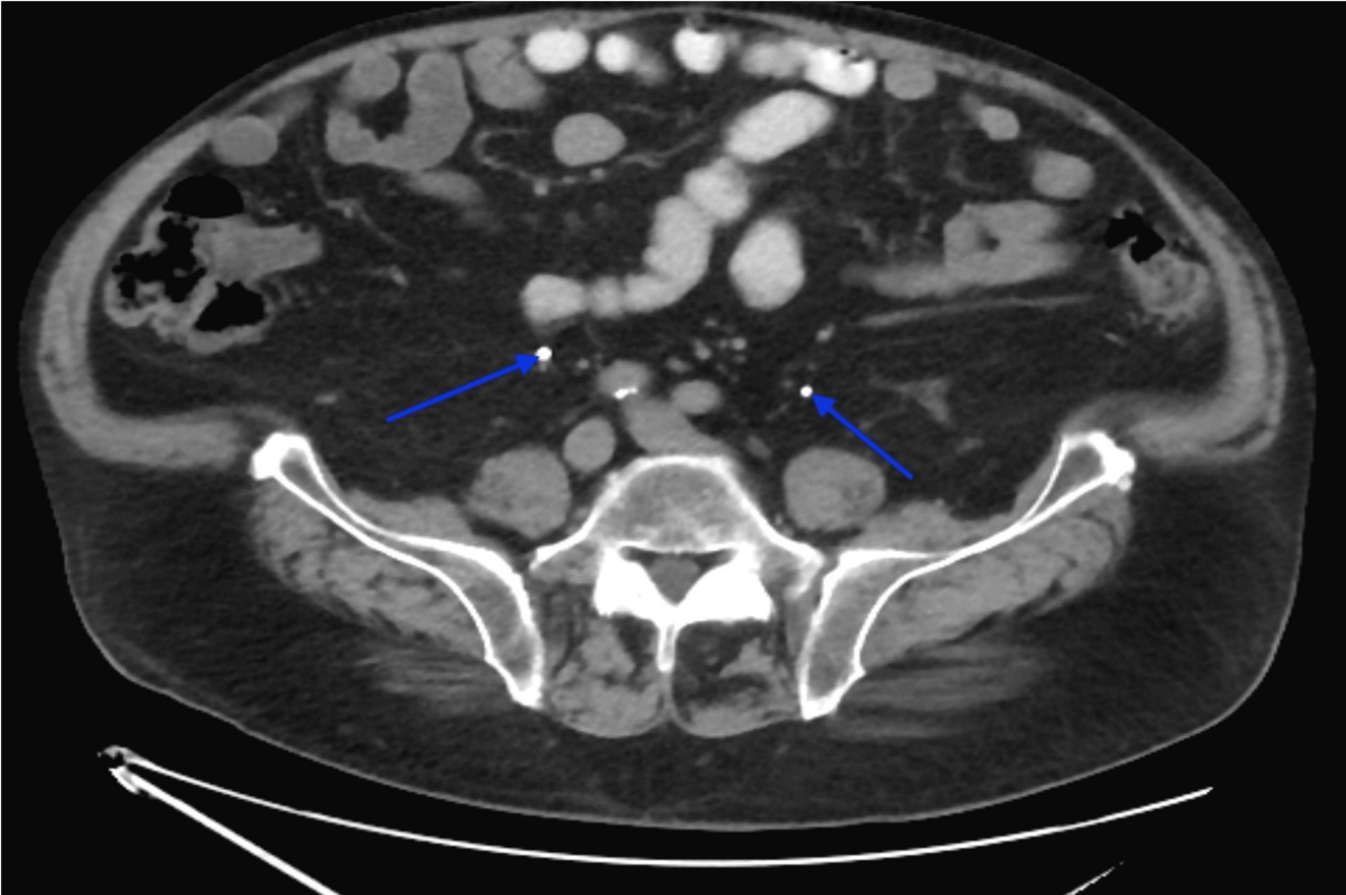
CT with contrast demonstrating significant anterior descent of patient's ureters (blue arrows) bilaterally within the abdomen.

**Figure 3:**
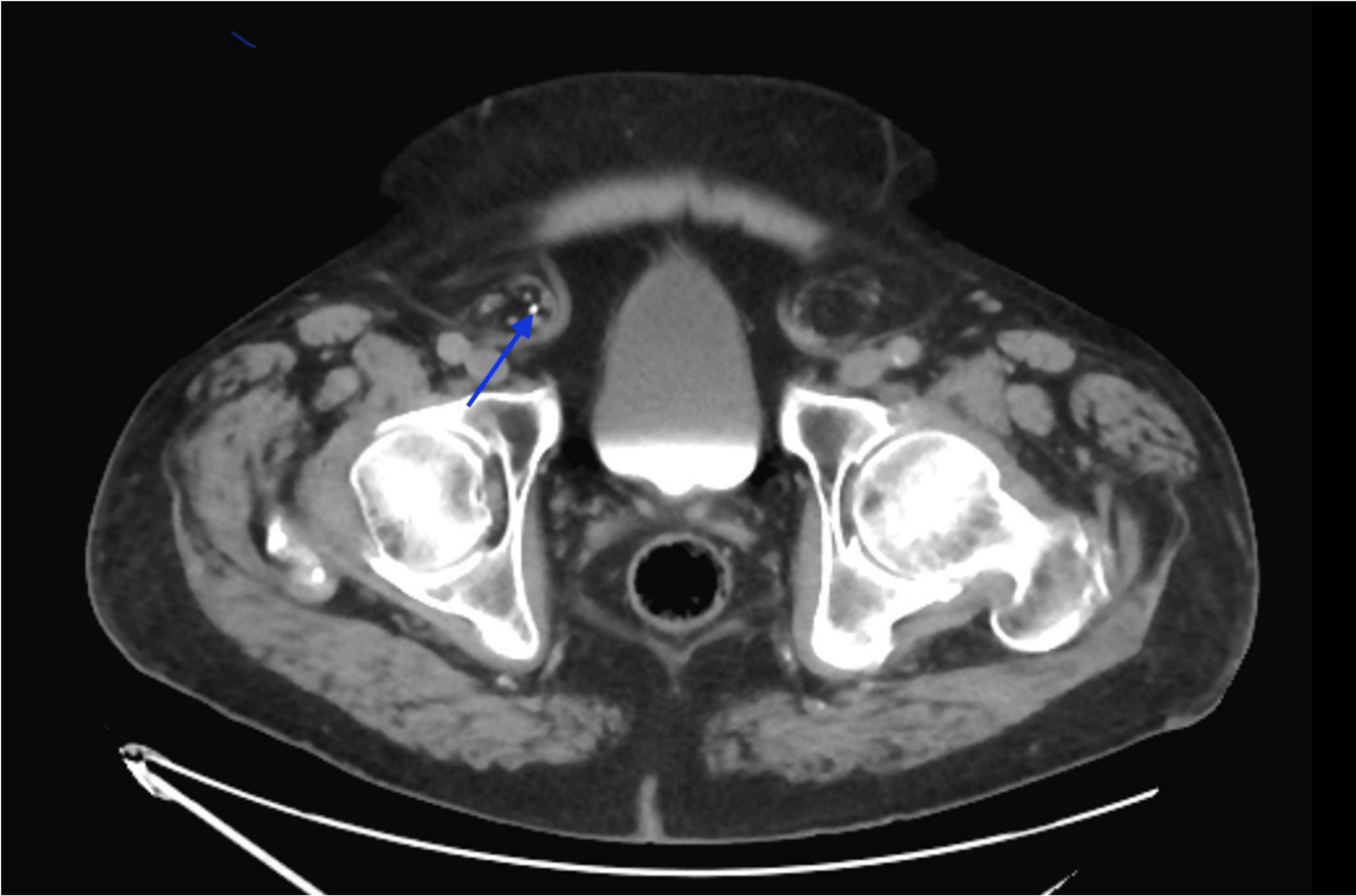
CT with contrast showing ureteral presence (blue arrow) within the inguinal hernia on the right. Still imaging made it difficult to properly display the left sided ureter.

A 76-year-old obese male presented to the Emergency Department with complaints of dark red stool slowly worsening over a one-week time span. The past medical history included atrial fibrillation, polycythemia vera, gastroesophageal reflux disease, insulin-dependent type 2 diabetes, hypertension, hyperlipidemia, hydrocele, overactive bladder, and untreated bilateral inguinal hernias. Additional history revealed that this patient survived multiple gunshot wounds during the Vietnam War, which were effectively stabilized at the time. The physical exam demonstrated an obese male with tenderness to palpation in the left lower abdominal quadrant. Reducible bulging tissues at the inguinal fold were present bilaterally. The patient received an abdominopelvic CT scan with and without contrast, where the principal diagnosis of diverticulitis was made. The ureters were nonobstructed at their origin near the renal pelvis, as represented in Figure 1. Of additional interest, the radiological impression also revealed that the ureteral course was found to be more anterior than expected, as shown in Figure 2, and passed within the inguinal hernias bilaterally, as shown in Figure 3. Additional findings from the CT scan demonstrated a 3 mm non-obstructing right renal calculus. Bilateral fat-containing inguinal hernias were identified, partially containing the lower ureters without evidence of obstruction.

The patient was admitted to the hospital and treated for diverticulitis with IV piperacillin/tazobactam 4.5 g every six hours for four days until the inflammation and bleeding resolved, and he was subsequently discharged with oral antibiotics for the remainder of the treatment course. The patient was then scheduled for an outpatient colonoscopy and prepped for discharge. He was informed of the presence of the ureters bilaterally in his previously diagnosed inguinal hernias and encouraged to continue conservative management. The patient was also informed of the possible risks of hernial incarceration/strangulation, urinary obstruction, as well as the potential risk of ureteral injury should he need surgery in the future.

## Discussion

Given that ureteral presence within an inguinal hernia is extraordinarily rare, this case further demonstrates the complexity of human anatomy due to the presence of this phenomenon bilaterally. With the increased morbidity and mortality risks involved in hernia repair in patients with this anatomical anomaly, the importance lies in early identification during preoperative imaging. This case highlights a potentially lucky, asymptomatic finding of this condition, which allowed for the proper steps and counseling to ensue.

This finding was unique in its nature of being asymptomatic, especially with bilateral presence. Although this condition may cause obstructive symptoms, the patient did not report dysuria or changes in urinary frequency during his hospital course or in his recent history. The insidious nature of this anatomical rarity further supports that patient symptoms should not be relied upon when determining the imaging workup for routine surgical procedures, such as herniorrhaphy. Because no two patient cases are identical, patients at high risk of complications (obesity, trauma history, etc.) should undergo a proper imaging workup prior to being taken to surgery. Although sources mention that a CT urogram is the best imaging modality to detect this phenomenon, it is expensive and should be reserved for patients with symptoms of urinary obstruction or pain [6]. Ultrasound, as a cheaper imaging modality, could serve as a routine substitute to be used in all patients with inguinal hernias. However, further clinical education on these rare cases is warranted among ultrasound users.

## Conclusions

Inguinal hernia repair is a common surgical procedure worldwide, making the presence of bilateral ureters within inguinal hernias a rare and notable finding. This condition can complicate an otherwise routine operation, especially in the absence of urinary obstruction, which highlights its insidious nature. This case underscores the importance of preoperative imaging in patients with a history of genitourinary obstruction, obesity, or significant trauma. Failure to identify a ureter within an inguinal hernia could represent a crucial mistake, as it increases the risk of operative injury and poses challenges even for experienced surgeons.
